# A case report of T cell/histiocyte-rich large B cell lymphoma misdiagnosed as lymphomatoid papulosis

**DOI:** 10.1097/MD.0000000000033407

**Published:** 2023-03-31

**Authors:** Taekwoon Kim, Jisung Kim, Joonsoo Park

**Affiliations:** a Department of Dermatology, School of Medicine, Daegu Catholic University, Daegu, Korea.

**Keywords:** lymphomatoid papulosis, T cell/histiocyte-rich large B cell lymphoma

## Abstract

T cell/histiocyte-rich large B cell lymphoma (THRLBCL) is an uncommon B cell lymphoma characterized by < 10% large neoplastic B cells in a background of abundant T cells and frequent histiocytes. If a skin lesion is the first clinical sign of lymphoma, the diagnosis might be difficult and misdiagnosed.

**Patient concerns:**

A 60-year-old woman presented with multiple erythematous umbilicated nodules on her left upper back for 3 months.

**Diagnoses:**

Through punch biopsy of the back lesion and additional excisional right inguinal lymph node biopsy, the patient was diagnosed with cutaneous metastasis of THRLBCL.

**Interventions:**

The patient was referred to the Hemato-oncology Department for chemotherapy.

**Outcomes:**

R-CHOP chemotherapy is currently in progress, and some skin lesions show improvement.

**Lessons:**

Skin lesions might be the first clinical sign of THRLBCL and when THRLBCL is suspected, careful further evaluation is essential for accurate diagnosis and treatment.

## 1. Introduction

T cell/histiocyte-rich large B cell lymphoma (THRLBCL) is an uncommon B cell lymphoma characterized by < 10% large neoplastic B cells in a background of abundant T cells and frequent histiocytes. ^[1,2]^ THRLBCL is a predominant nodal neoplasm, with or without cutaneous involvement. Although extranodal sites can be involved, an exclusively extranodal presentation, including primary cutaneous lymphoma, at diagnosis is extremely rare.^[3,4]^ Therefore, when THRLBCL is suspected for the first time through skin lesions, careful evaluation is essential for accurate diagnosis. This report discusses a rare case of THRLBCL with cutaneous involvement, which was first misdiagnosed as lymphomatoid papulosis.

## 2. Case report

**Figure 1.**
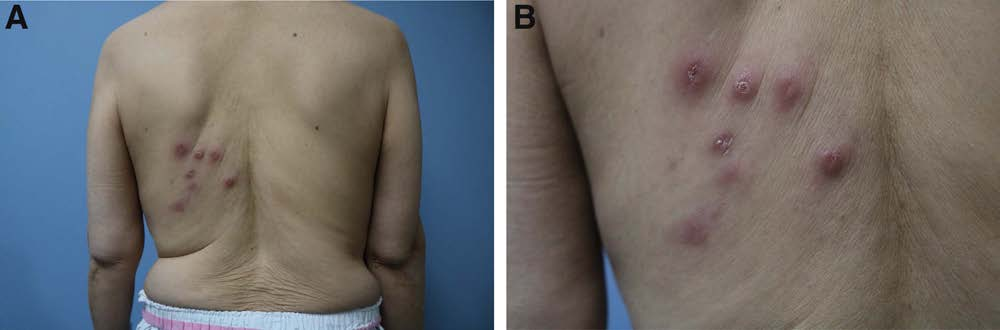
(A, B) Clinical presentation of multiple erythematous umbilicated nodules on the patient’s left upper back.

A 60-year-old woman presented with multiple erythematous umbilicated nodules on the left upper back. The patient reported that she first noticed the lesion 3 months previously and that it later increased in size and number. There were no symptoms, such as pain or pruritus. The patient had a medical history of rheumatoid arthritis for 10 years and no specific family history. Physical examination revealed multiple non-tender erythematous umbilicated nodules on the left upper back (Fig. 1A and B). In addition, a single 1.5 cm-sized non-tender subcutaneous mass was found in the right inguinal area. The patient reported that the lesion had gradually increased in size over 10 months. There were no obvious abnormalities in routine blood tests, coagulation, or liver or kidney function.

**Figure 2.**
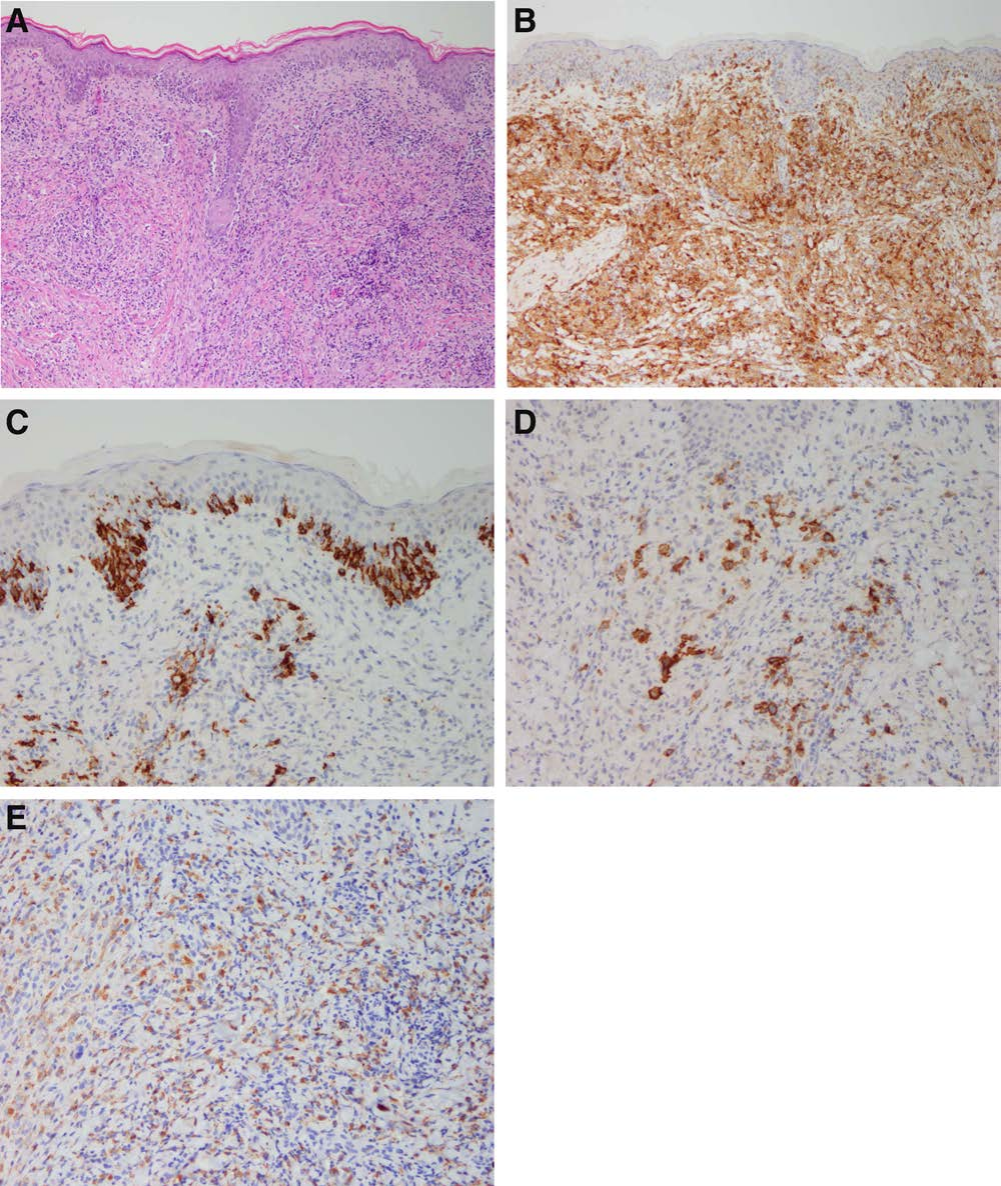
Histological features of punch biopsy on the left upper back. (A) Diffuse lymphocytes infiltration in the dermis (H&E, × 100). (B) Lymphocytes infiltration diffusely positive for CD4 (CD4, × 100). (C) Immunohistochemical staining showing large cells positive for CD20 (CD20, × 200). (D) Immunohistochemical staining showing large cells weakly and focally positive for CD30 (CD30, × 200). (E) Numerous CD68-positive histiocytes in the background (CD68, × 200).

**Figure 3.**
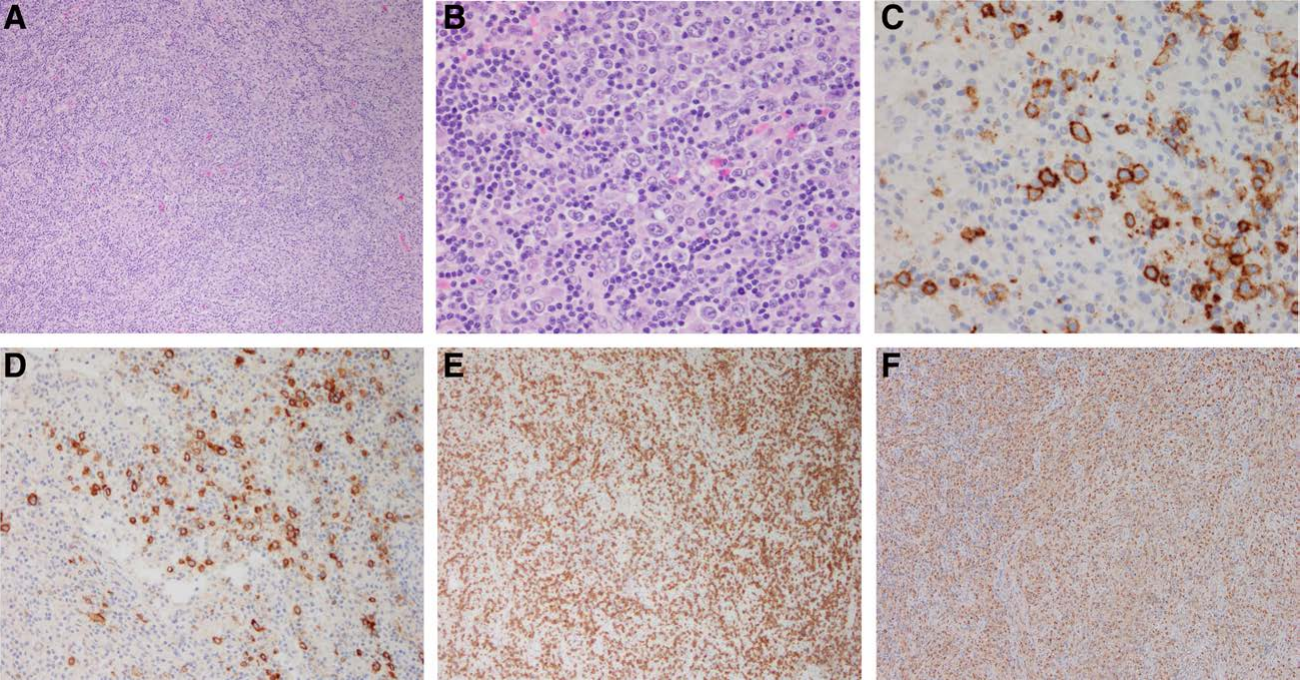
Histological features of right inguinal lymph node excisional biopsy. (A) Diffuse lymphocyte infiltration (H&E, × 100). (B) Scattered large atypical cells in a background rich in small lymphoid cells (H&E, × 400). (C) Immunohistochemical staining showing large atypical cells positive for CD20 (CD20, × 400). (D) Immunohistochemical staining showing some large atypical cells positive for CD30 (CD30, × 200). (E) Numerous CD3-positive small cells in the background (CD3, × 100). (F) Numerous CD68-positive histiocytes in the background (CD68, × 100).

A punch biopsy was performed on the left upper back, and histologically, diffuse lymphocyte infiltration was observed in the superficial, deep dermis, and subcutaneous layers (Fig. 2A). Immunohistochemical staining revealed that these lymphocytes were diffusely positive for CD4 (Fig. 2B), some large cells were positive for CD20, and weakly and focally positive for CD30 (Fig. 2C and D). Some small cells positive for CD3 surrounded large cells. Additionally, some small cells were positive for CD68 (Fig. 2E). In terms of some cells that were positive for CD4 and CD30, including clinical manifestations, there was a possibility of lymphomatoid papulosis. However, in some cells positive for CD20 and some surrounding cells positive for CD68, including diffuse lymphocyte infiltration in the dermis and subcutaneous layer, there is a possibility of other lymphomas and cutaneous metastases of other lymphomas. Therefore, the diagnosis was difficult. A single 1.5 cm-sized subcutaneous mass was found in the right inguinal area. An inguinal lymph node excisional biopsy was performed in our General Surgery Department. Similar to the biopsy of the back, histologically diffuse lymphocyte infiltration was observed (Fig. 3A). There were a few scattered large atypical cells with pleomorphic vesicular nuclei and prominent nucleoli (Fig. 3B). In addition, numerous small lymphoid cells surrounded large atypical cells. Immunohistochemical staining revealed that these atypical large cells were positive for CD20, and some cells were weakly and focally positive for CD30 (Fig. 3C and D). In addition, these large atypical cells were positive for Bcl-2 and negative for CD10, CD15, and EBV. Most small lymphoid cells were CD3 positive (Fig. 3E). Some cells were CD68 positive histiocytes (Fig. 3F). Overall, histologically similar patterns were observed in comparison with the biopsy on the back. The molecular analysis demonstrates immunoglobulin heavy-chain gene rearrangement. Based on these findings, a diagnosis of T cell/histiocyte-rich large B cell lymphoma was established. Complete staging, including computed tomography and positron emission tomography, was performed, and the involvement of lymphoma was identified, including the skin and subcutaneous tissue of the chest wall, both lungs and both thighs. Finally, the patient was diagnosed with cutaneous metastasis of T cell/histiocyte-rich large B cell lymphoma and referred to the Hemato-oncology Department for further evaluation and chemotherapy.

## 3. Discussion

THRLBCL, previously considered as an uncommon variant of diffuse large B cell lymphoma, has been classified by the World Health Organization as a separate entity since 2008. THRLBCL is characterized by < 10% large neoplastic B cells in a background of abundant T cells and histiocytes.^[1]^ The neoplastic B cells usually represent single cells.

THRLBCL is uncommon, with an incidence of 0.23/million individuals. It mostly affects middle-aged male patients, and the sex ratio is almost 3:1.^[2]^ THRLBCL is a predominant nodal neoplasm, with or without cutaneous involvement. However, extranodal sites can be involved, and the most common extranodal sites are the liver, spleen, bone marrow, and lungs.^[5]^ Although extranodal sites can be involved, exclusive extranodal presentation at the time of diagnosis is extremely rare.^[4]^ Primary cutaneous THRLBCL is extremely rare. Therefore, as in this case, further evaluation is essential when THRLBCL is suspected for the first time in the skin. THRLBCL is often disseminated at the time of diagnosis because more than half of the patients present with the disease at an advanced stage.^[2]^ This the poor outcome might be related to the advanced stage of the disease.^[6]^

Histologically, neoplastic cells have centroblastic, immunoblastic, or Hodgkin-like morphology.^[2]^ Immunohistochemical staining showed that the neoplastic B cells expressed pan-B cell markers, such as CD20 and CD79a, but were negative for CD5, CD15, and CD138; neoplastic B cells rarely express CD30. Tumor cells are typically positive for Bcl-6 and negative for CD10. Bcl-2 expression was positive in up to 50% of cells. The background environment is composed of lymphocytes positive for CD3, CD5, or CD45RO, which might be positive for CD4 but mostly positive for CD8. There may be CD68-positive histiocytes surrounding the tumor cells.^[1]^ In the present patient, neoplastic B cells were positive for CD20 and Bcl-2 and weakly and focally positive for CD30. Reactive background infiltrates were positive for CD3 and CD68. The immunohistochemical findings in the present case are essential for the diagnosis of this rare lymphoma. An additional biopsy through a careful physical examination showed a histological pattern similar to the first biopsy, and these findings led to an accurate diagnosis.

Owing to the low fraction of neoplastic cells, molecular analysis is generally challenging in THRLBCL.^[2]^ In the present case, the molecular analysis demonstrated immunoglobulin heavy-chain gene rearrangement, which was helpful in the diagnosis.

The most commonly recommended treatment for THRLBCL is CHOP chemotherapy.^[3]^ Recently, a combination of rituximab (R-CHOP) was used. R-CHOP chemotherapy appears to be helpful for the treatment of THRLBCL, and combined radiotherapy may be considered.^[7]^ Appropriate initial treatment may result in outcomes similar to those of conventional diffuse large B cell lymphoma treatment.^[6]^

In summary, the diagnosis of THRLBCL through skin biopsy for the first time is difficult. THRLBCL, presenting primarily on the skin, is extremely rare and can be misdiagnosed as a different disease. However, skin lesions might be the first clinical sign of THRLBCL, and careful evaluation is essential for accurate diagnosis and treatment.

## Author contributions

**Conceptualization:** Taekwoon Kim, Jisung Kim.

**Data curation:** Taekwoon Kim, Jisung Kim.

**Supervision:** Joonsoo Park.

**Writing – original draft:** Taekwoon Kim, Jisung Kim, Joonsoo Park.

**Writing – review & editing:** Taekwoon Kim, Jisung Kim, Joonsoo Park.
